# Assessing the Suitability of Elite Lines for Hybrid Seed Production and as Testers in Wide Crosses With Wheat Genetic Resources

**DOI:** 10.3389/fpls.2021.689825

**Published:** 2021-06-14

**Authors:** Johannes Schneider, Marcel O. Berkner, Norman Philipp, Albert W. Schulthess, Jochen C. Reif

**Affiliations:** Leibniz Institute of Plant Genetics and Crop Plant Research (IPK), Seeland, Germany

**Keywords:** hybrid seed production, female receptivity, wheat, hybrid breeding, flowering biology

## Abstract

The use of genetic resources in breeding is considered critical to ensure future selection gain, but the absence of important adaptation genes often masks the breeding value of genetic resources for grain yield. Testing genetic resources in a hybrid background has been proposed as a solution to obtain unbiased estimates of breeding values for grain yield. In our study, we evaluated the suitability of European wheat elite lines for implementing this hybrid strategy, focusing on maximizing seed yield in hybrid production and reducing masking effects due to susceptibility to lodging, yellow rust, and leaf rust of genetic resources. Over a 3-year period, 63 wheat elite female lines were crossed with eight male plant genetic resources in a multi-environment field experiment to evaluate seed yield on the female side. Then, the resulting hybrids and their parents were tested for plant height, lodging, and susceptibility to yellow rust and leaf rust in a further field experiment at multiple locations. We found that seed yield was strongly influenced by the elite wheat line choice in addition to environment and observed substantial differences among elite tester lines in their ability to reduce susceptibility to lodging, yellow rust, and leaf rust when the hybrid strategy was implemented. Consequently, breeders can significantly increase the amount of hybrid seed produced in wide crosses through appropriate tester choice and adapt genetic resources of wheat with the hybrid strategy to the modern cropping system.

## Introduction

Wheat (*Triticum aestivum* L.) is one of the most important crops for the world’s food supply, with an estimated production of around 762.2 million tons in 2019/2020 (FAO, 2020). To feed a world population of ∼9 billion, wheat yields must be increased by 1.8% annually by 2050 (Godfray et al., 2010; Ray et al., 2013). Yet, the average annual increase in wheat yields is currently 0.9% (Ray et al., 2013). In addition, the combination of restricted availability of further fertile arable land and limitation of fertilization further challenges the production of wheat. Moreover, the consequences of climate change with the associated loss of biodiversity in agricultural landscapes will pose challenges to global food production which might even be severed by socio-economic circumstances in developing countries (Godfray et al., 2010; Tilman et al., 2011). It was therefore hypothesized that increasing agricultural production is a promising solution to minimize the growing threat of a global food crisis (Tilman et al., 2011; Ray et al., 2013). Particularly in wheat, varieties with a high yield potential and better tolerance to biotic and abiotic stresses need to be bred.

Wheat breeding has been based so far on a limited population of elite genotypes. For instance, the estimated effective population size of a Central European breeding program amounted to 31 individuals (He et al., 2017). In order to adapt wheat to future requirements, the increased use of plant genetic resources is therefore proposed (Khush, 2013; Rasheed et al., 2017; Mascher et al., 2019). Regional landraces, local varieties and species related to wheat are sources of genetic variation and can be carriers of favorable alleles. Through their discovery, they can help to diversify wheat breeding (Feuillet et al., 2008). Many of these wheat accessions are preserved in various gene banks around the world (Bonjean and Angus, 2001). Nevertheless, finding beneficial allele combinations, and integrating them into pre-breeding programs is difficult and very time-consuming (McCouch et al., 2013). One reason for this is that the phenotyping of many plant genetic resources in field trials is very complex and expensive (Montes et al., 2007). On the other hand, the breeding potential for important traits, such as grain yield, is hidden by unfavorable genetic background effects of non-adapted genetic resources that present limitations like susceptibility to lodging and diseases (Longin et al., 2014).

In order to test wheat genetic resources for their breeding values for grain yield, Longin and Reif (2014) proposed to implement a hybrid strategy. The breeding value is in this case not evaluated by phenotyping the *per se* performance of the plant genetic resources, but rather by estimating their general combining ability in a hybrid background when crossed to an adapted elite line. Crosses of plant genetic resources with elite varieties has already been applied in other crops such as rye (*Secale cereale* L.) (Fischer et al., 2010), maize (*Zea mays* L.) (Böhm et al., 2014), barley (*Hordeum vulgare* L.) (Sommer et al., 2019), and sorghum (Elagib, 2007). These studies were based on crosses between a limited number of elite lines and have not investigated the optimum choice of a tester. Despite this, the studies indicated that the genetic base of heterotic pools can be broadened with plant genetic resources, which can increase the selection gain for grain yield in hybrid breeding.

The implementation of the hybrid strategy in wheat can be enabled, for example, by using chemical hybridization agents (Kempe et al., 2014). Due to plant height and flowering characteristics, wheat genetic resources can be used predominantly as males and elite lines as females in hybrid seed production. In this case, selected plant genetic resources should tend toward open pollination – a property that has been observed earlier for wheat genetic resources of the *German Federal ex situ Genebank for Agricultural and Horticultural Crops* (Boeven et al., 2020).

For a successful application of the hybrid strategy in wheat, finding suitable elite female tester lines is crucial, as these lines increase the efficiency of hybrid seed production. Boeven et al. (2018) reported that the quantity of hybrid seed also depends on the female line itself. Therefore, a suitable tester line should allow a high seed yield in hybrid seed production. In addition, a suitable tester line should correct for masking effects of the grain yield potential such as susceptibility to disease and lodging susceptibility. Experimental studies on the selection of suitable testers to implement the hybrid strategy for mining valuable diversity in wheat genetic resources have not yet been conducted.

In this study, the objectives were to (1) examine a diverse set of European wheat elite lines for their suitability in hybrid seed production in wide crosses with plant genetic resources, (2) investigate phenotypically the mode of inheritance of female lines in the hybrid background for the traits lodging tolerance, plant height, and resistance to yellow rust and leaf rust, and (3) suggest suitable elite tester lines to implement the hybrid strategy for wheat genetic resources.

## Materials and Methods

### Plant Material

Our study was based on 72 winter wheat lines (Table 1 and Supplementary Table 1). From the total set of lines, 63 served as female lines and eight as male lines in hybrid seed production. The female lines represented a selection of modern elite varieties bred for wheat production in Europe. The male lines were plant genetic resources hosted at the *Federal ex situ Gene Bank for Agricultural and Horticultural Crop Species* of Germany. The male lines were selected for their excellent pollination properties demonstrated in previous hybrid wheat trials (Boeven et al., 2020), as well as their synchronization of flowering time and plant height with the female lines. The female and male lines were divided into four flowering time classes: very early, early, medium and late.

**TABLE 1:** Description of plant genetic resources used as male parents in hybrid seed production experiment.

Accession	Botanical Name	DOI	Origin	Flowering time	Plant height (cm)	Lodging tolerance	Strip rust resistance	Leaf rust resistance
TRI_9670	*Triticum aestivum L. var. aestivum*	10.25642/IPK/GBIS/9642	Bulgaria	Very early	92,81	1	4	3
TRI_9326	*Triticum aestivum L. var. lutescens (Alef.) Mansf.*	10.25642/IPK/GBIS/9298	Germany	Late	91,81	1	2	4
TRI_8280	*Triticum aestivum L. var. lutescens (Alef.) Mansf.*	10.25642/IPK/GBIS/8252	Netherlands	Medium	141,80	4	2	4
TRI_6747	*Triticum aestivum L. var. lutescens (Alef.) Mansf.*	10.25642/IPK/GBIS/6747	Sowjet Union	Very early	98,34	2	2	2
TRI_5082	*Triticum aestivum L. var. lutescens (Alef.) Mansf.*	10.25642/IPK/GBIS/5082	Sowjet Union	Late	146,48	4	3	4
TRI_13344	*Triticum aestivum L. var. lutescens (Alef.) Mansf.*	10.25642/IPK/GBIS/13311	Unknown	Early	93,14	1	3	4
TRI_13141	*Triticum aestivum L. var. lutescens (Alef.) Mansf.*	10.25642/IPK/GBIS/13109	Unknown	Medium	109,45	2	4	4
TRI_10384	*Triticum aestivum L. var. lutescens (Alef.) Mansf.*	10.25642/IPK/GBIS/10356	France	Early	118,36	3	2	3

### Assessing the Hybrid Seed Set in a Multi-environment Field Experiment

To study the seed yield of the female lines in hybrid seed production, a non-replicated field experiment was carried out in three environments (Supplementary Table 2 and Supplementary Figure 1). In this experiment, each female was crossed with two male lines of the corresponding flowering time class. In 2016/2017 and 2017/2018, the field trial included 100 and in 2018/2019 116 single-crosses, whereby 50 and 58 female lines were crossed with the corresponding two male lines, respectively. The crosses of each male line were spatially isolated, resulting in eight crossing blocks. The male and female lines were grown on 7.5 m^2^ plots in the year 2016/2017 and on 13 m^2^ plots in the following years. Male and female plots were sown side by side to facilitate controlled pollination. To prevent cross-pollination by other pollinators, each crossing block had an additional isolation with triticale or cytoplasmic male sterile rye. The sowing intensities were 300 kernels per m^2^ for the female lines and 150 kernels per m^–2^ for the male lines. Female lines were emasculated using the chemical hybridizing agent Clofencet, which chemically suppresses pollen development (Parodi and de los Angeles Gaju, 2009). The application took place twice in a period from the end of April to mid-May in order to hit the main and secondary shoots of the individual wheat plants with the gametocide. The specific application date for each female plot was determined by the length of the ear in the stem at the time of the stem extension. For the first application with the gametocide the wheat ears in the main shoot should have a length of 2.5 cm. The second treatment was carried out 6 to 7 days after the first application.

Sterility level and flowering time were assessed for each genotype in the hybrid seed production trial. Sterility level was determined after the last gametocide treatment, by isolating the main and secondary shoots of three wheat plants in each treated plot with bags. The bags were placed at the beginning, at the end, and in the middle of a plot. For each bag the number of kernels were counted and summed for each plot. Flowering time was recorded in days after the 1^*st*^ of January as (1) the start of flowering, (2) the peak of flowering time, and (3) the end of flowering. The plots of each female line were harvested by using a plot combine. After harvest, the seeds were processed and the seed yield per plot at 14% moisture content was assessed.

### Analyses of the Hybrid Seed Yield Field Experiment

The data on sterility and flowering time were analyzed fitting following linear mixed model:

(1) $${\text{y}}_{\text{ij}}=\mu +{\text{g}}_{\text{i}}+{\text{e}}_{\text{j}}+{\text{g}}_{\text{i}}{\text{e}}_{\text{j}}+\varepsilon_{\text{ij}}.$$

Here, *y*_*ij*_ refers to the observed phenotypic value of the *i*-th genotype in the *j*-th environment, *μ* is the total mean value, *g*_*i*_ is the effect of the *i*-th genotype, *e*_*j*_ is the effect of the *j*-th environment, *g_*i*_e_*j*_* is the effect of the interaction between the *i*-th genotype and the *j*-th environment, and ε_*ij*_ is the residual error from the model for the observed phenotypic value. With the exception of μ, all effects were modeled as random to estimate the variance components. In addition, genotypes were modeled as fixed effects to obtain the Best Linear Unbiased Estimations (BLUEs). Outlier tests were performed using the χ^2^-test as described by Wilks (1938) and Chernoff (1954).

For the evaluation of the hybrid seed production experiment, only the cross-pollinated female lines should be considered and a potential bias due to selfing has to be reduced. We therefore removed all plot values from the data set, where, the chemical emasculation had not worked properly. This was implemented by considering the number of kernels m^–2^ in the isolation bags for each plot. The 85% percentile Q_0.85_ served as threshold. The resulting high-quality data was used to fit Equation (1). Broad-sense heritability was determined for all traits as:

(2) $${\text{h}}^{2}=\frac{\sigma_{g}^{2}}{\sigma_{g}^{2}+\frac{\sigma_{g\times e}^{2}}{E}+\frac{\sigma_{\varepsilon }^{2}}{E*R}},$$

where, $\sigma_{\text{g}}^{2}$ refers to the variance of the genotype. $\sigma_{\text{g}\times \text{e}}^{2}$ stands for the variance of the interaction between the genotype and the environment and $\sigma_{\varepsilon }^{2}$ is the error variance. Furthermore, *E* and *R* refers to the number of environments and replicates, respectively.

Moreover, the following model was used to analyze the seed yield of the female lines:

(3) $$y_{ijk}=\mu +f_{i}+m_{j}+f_{i}m_{j}+e_{k}+f_{i}e_{k}+m_{j}e_{k}+\varepsilon_{ijk}.$$

Here, *y*_*ijk*_ is the observed phenotypic value of the *i*-th female when crossed with the *j*-th male in the *k*-th environment. μ refers to the mean value of the population, *f*_*i*_ to the effect of the *i*-th female, *m*_*j*_ to the *j*-th male, *f_*i*_m_*j*_* to the interaction effect between the *i*-th female and the *j*-th male, *e*_*k*_ to the effect of the *k*-th environment, *f_*i*_e_*k*_* to the effect of the interaction between the *i*th female and the *k-*th environment, *m_*i*_e_*k*_* to the effect of the interaction between the *j*-th male and the *k*-th environment, and *ε_*ijk*_* refers to the residual. Excepting μ, all effects were modeled as random.

The narrow-sense heritability of the female lines was estimated for seed yield as:

(4) $$h^{2}=\frac{\sigma_{f}^{2}}{\sigma_{f}^{2}+\frac{\sigma_{m}^{2}}{M}+\frac{\sigma_{f\times m}^{2}}{C}+\frac{\sigma_{f\times e}^{2}}{E}+\frac{\sigma_{\epsilon }^{2}}{E*R}}.$$

Here, $\sigma_{\text{f}}^{2}$ stands for the genetic variance of the females and $\sigma_{\text{m}}^{2}$ for the genetic variance of the males, $\sigma_{\text{f}\times \text{m}}^{2}$ refers to the variance of the interaction between the female lines and the male lines and $\sigma_{\text{f}\times \text{e}}^{2}$ to the variance of the interaction between the female lines and the environment, while $\sigma_{\epsilon }^{2}$ is the variance of the residuals. *M, C, E*, and *R* stand for the number of males, crosses, environments, and replicates, respectively.

### Field Evaluation of the Hybrids and Their Parental Lines

In total 100 hybrids as well as their 50 female and 8 male parental lines were evaluated in replicated field trials at four environments in Germany: Gatersleben (51° 49′ 23° N, 11° 17′ 13° E) and Quedlinburg (51°47′ 15.6 ′′N 11°08′ 36.3′′ E) in the growing seasons 2017/2018 and 2018/2019. The experimental design was an alpha lattice design with ten plots per incomplete block. The experimental plot comprised two rows of plants and was 0.8 m long. No plant protection or fertilization measures were applied.

Five traits were determined: Hybridity, plant height, lodging tolerance, as well as resistance to yellow rust and leaf rust. Hybridity was visually scored in % at BBCH (Obst and Gehring, 2002) stages 59, 69, and 85 (Zadok stage 59, 69, 85) considering the uniformity of the stands as well as the phenotype of the parental lines. Plant height was measured in cm after the end of flowering (BBCH stage 69; Zadok stage 69). Lodging tolerance was assessed at the time of maturity (BBCH stage 97, Zadok stage 97) using a scale from 1 (no lodging) to 9 (whole plot was lodging). Leaf rust and yellow rust was scored at the date of flowering (BBCH stage 65, Zadok stage 65) on the flag leaf. An ordinal scale from 1 to 9 on the basis of the Bundessortenamt. (2000) was used for this two traits, where, one stands for minimal symptoms and nine indicates extensive disease symptoms.

All hybrids with a hybridity below 75% were not considered for further analysis. Moreover, we performed an outlier test following Anscombe and Tukey (1963). The high-quality phenotypic data was used and following model was fitted:

(5) $$\begin{array}{c} y_{ijkmn}=\mu +f_{i}+m_{j}+f_{i}m_{j}+e_{k}+f_{i}e_{k}+m_{j}e_{k}+r_{m}+\\ b_{n}+\varepsilon_{ijkmn}. \end{array}$$

Here, *y*_*ijkm*_ stands for the observed phenotypic value of a hybrid resulting from a crossing of an *i*-th female with a *j*-th male in a *k*-th environment from an *m*-th replication in an *n*-th block. μ is the mean value of the population, *f*_*i*_ stands for the effect of the *i*-th female, *m*_*j*_ is the effect of the *j*-th male, *f_*i*_m_*j*_* is the effect of the interaction between the *i*-th female and *j*-th male, *e*_*k*_ is the effect of the *k*-th environment, *f_*i*_e_*k*_* is the effect of the interaction between the *i*-th female and the *k*-th environment, *m_*j*_e_*k*_* is the interaction between the *j*-th male and the *k*-th environment, *r*_*m*_ stands for the *m*-th replication, *b*_*n*_ is the effect of the *n*-th block nested within replications, and *ε_*ijkmn*_* stands for the residual error.

The best linear unbiased estimations (BLUEs) for each genotype across the environments, replications and blocks were obtained using the following model:

(6) $$y_{ijkm}=\mu +g_{i}+e_{j}+g_{i}e_{j}+r_{k}+b_{m}+\varepsilon_{ijkm}.$$

Here, *y*_*ijkm*_ is the observed phenotypic value of the *k*-th repetition of the *i*-th genotype in the *m*-th block on the *j-*th environment, *g*_*i*_ is the effect of the *i*-th genotype, *e*_*j*_ is the effect *j*-th environment, *g_*i*_e_*j*_* is the interaction of the *i*-th genotype with the *j*-th environment, *r*_*k*_ is the effect of the *k*-th environment, *b*_*m*_ is the effect of the *m*-th block and *ε_*ijkm*_* is the residual. For the calculation of the BLUEs, the effect of the genotype was defined as fixed and the remaining effects as random. All mixed model equations were fitted using the ASReml V 4 R package (Butler et al., 2018), while all computational methods were implemented in R environment version 3.6.1. (R Core Team, 2020).

## Results

### Data Curation Reduced Confounding Effects of Partial Fertility

The sterility level of females in the production experiment was determined by counting the number of kernels in insolation bags. Interestingly, for the raw data, we found that the genetic variance of sterility was significantly different from zero (*P* < 0.001), resulting in a heritability of 0.32 (Table 2). This clearly highlighted the need for data curation to avoid confounding effects of partial fertility on female seed yield in hybrid seed production. Elimination of all plots with a sterility value above the 85% quantile resulted in estimates of genetic variance and heritability of zero. Thus, after correction for outliers, there was no significant genotypic variation within the female lines for sterility and this trait was dependent only on environments, which generated an appropriate basis to investigate the seed yield in the hybrid production experiment.

**TABLE 2:** Variance components underlying phenotypic variation of the sterility level and its broad-sense heritability (h^2^) using raw and curated data of the hybrid seed production experiment.

Source	Raw data	Curated data^*a*^
Number of genotypes	68	63
Number of environments	3	3
σ^2^_*e*_	75.56**	69.74**
σ^2^_*g*_	10.52***	0.00
σ^2^_*g* × e_	20.61***	0.81
σ^2^_ε_	70.09	20.59
h^2^	0.32	0.00

Phenotypic variation was decomposed into environments (σ^2^_e_), genotypes (σ^2^_g_), their interactions (σ^2^_g × e_), and the residuals (σ^2^_ε_). ^a^Curated data through elimination of plots with sterility value surpassing the 85% quantile. ** and *** significantly different from zero with P < 0.01 and 0.001, respectively.

### Flowering Time Was Properly Synchronized and Affected the Seed Yield Only Marginally

**FIGURE 1:**
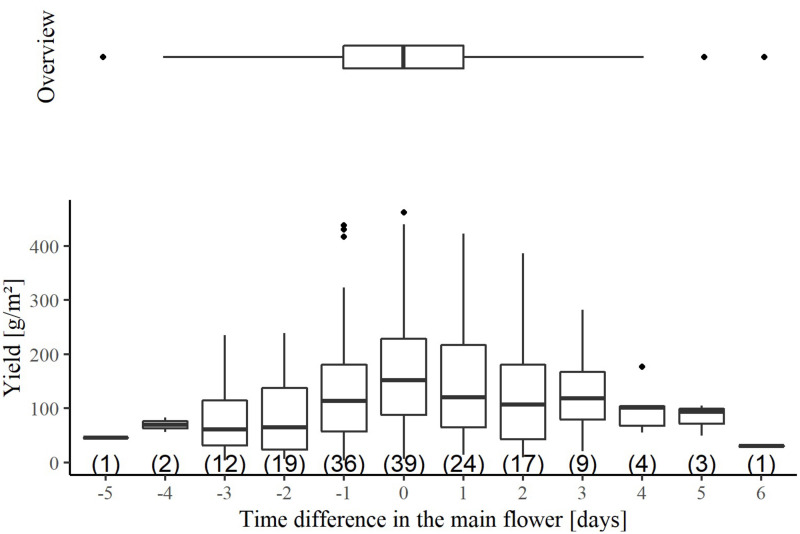
The association between the hybrid seed yield of the female lines and the time difference of the main flowering between the crossing partners in the hybrid seed production experiment. The temporal difference results from subtracting the time of the main flowering of the male and the female. Negative values indicate that the main flowering of the male was earlier than that of the female. Conversely, a positive time difference stands for a later flowering of the male than the female. The horizontal boxplot shown above, summarizes the total variation for the time difference in main flowering. The number of observations per boxplot is given in parenthesis.

The flowering time of the individual wheat lines was documented for all plots in the production experiment (Supplementary Figure 2). It is noticeable that the wheat lines reached the peak of flowering 10 days earlier in the 2017/2018 season than in the other two seasons. To achieve fertilization, the flowering periods of the males must overlap with those of the females, which was successfully realized for all cross combinations (Supplementary Figure 2). The peak of flowering time of females was between 6 days earlier to 5 days later than their crossing partners (Figure 1). Nevertheless, more than half of the cross combinations were in a range, where, the peak of flowering of the female line was 1 day earlier to1 day later than that of the male line. In addition, the time difference in the peak of flowering explained only 9% of the variation in seed yield of the female line. Thus, the crossing design resulted in a proper synchronization of flowering time.

### Seed Yields Were Strongly Influenced by the Environment and the Female Lines

**FIGURE 2:**
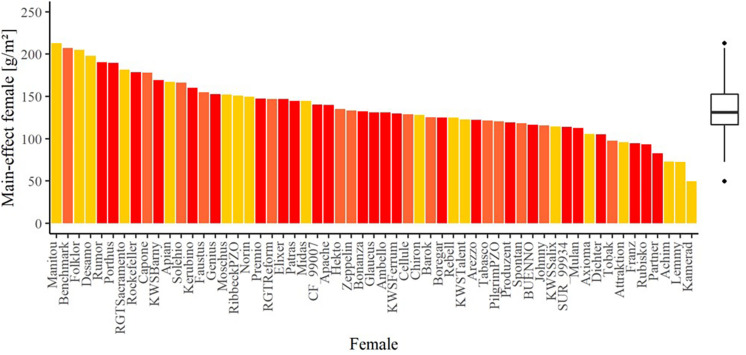
The main effect of the female lines for the trait seed yield in the hybrid seed production experiment. The colors indicate the number of test years: dark red bars: three years, orange bars: 2 years, and yellow bars: 1 year. The boxplot shows the summary over all female lines.

The average seed yield was highest in 2018/2019 with 274.20 g per m^2^ and lowest in 2017/2018 with 71.43 g per m^2^ (Supplementary Figure 3). Despite the pronounced difference, the correlation for the hybrid seed yield on the females between the trial years is high (Supplementary Table 3) and thus, values of all years were considered for the analysis. Accordingly, 65% of the total seed yield variation was explained by the environments, followed by the female lines explaining 11% of the total phenotypic variation (Table 3). The estimated effects of female lines on seed yield in hybrid production varied widely with more than a five-fold difference between the minimum of 49.53 g per m^2^ for *Kamerad* and maximum value of 212.82 g per m^2^ for *Manitou* (Figure 2). These two mentioned varieties were, however, just tested in 1 year. Focusing exclusively on female lines which were tested in multiple years, the estimated effects ranged from the minimum of 82.61 g per m^2^ for *Partner* to the maximum value of 207.08 g per m^2^ for *Benchmark*. The effect of the male lines explained only about 5% and their interaction effects with female lines explained 0.62% of the total phenotypic variance for seed yield, respectively (Table 3). The heritability of the seed yield was high and amounted to 0.75. As yield is influenced by the environment, only the varieties, that had been tested for more than 2 years, could be considered for the tester selection.

**TABLE 3:** Variance components and narrow-sense heritability (h^2^) for seed yield (g m^–2^) in the hybrid seed production experiment.

Source	Seed yield
Number of environments	3
Number of females	4.25
Number of males	57
σ^2^_*e*_	9580.44***
σ^2^_*f*_	1709.67***
σ^2^_*m*_	860.77***
σ^2^_*f x m*_	91.20*
σ^2^_*f x e*_	398.83***
σ^2^_*m x e*_	646.34***
σ^2^_ε_	1408.94
h^2^	0.75

Phenotypic variation was decomposed into environments (σ^2^_e_), females (σ^2^_f_), males (σ^2^_m_), the interactions between female and males (σ^2^_f × m_), their interactions with environments (σ^2^_f × e_ and σ^2^_m × e_) and the residuals (σ^2^_ε_) * and *** significantly different from zero with P < 0.05 and 0.001, respectively.

### Female Lines Differed in Their Mode of Inheritance for Plant Height, Lodging Tolerance, and Disease Resistance

We evaluated the performance of the female and male parental lines and the resulting single-cross hybrids in a multi-environmental field experiment for the traits plant height, lodging tolerance, and resistance to yellow rust and leaf rust. Heritability estimates were moderate to high with a range from 0.50 for leaf rust resistance to 0.92 for plant height (Supplementary Table 4). Interestingly, plant height was only correlated to lodging tolerance with *r* = 0.72 (*P* < 0.05). Therefore, in the following, we considered only the three traits lodging tolerance, yellow rust resistance, and leaf rust resistance in selecting of appropriate elite tester lines to improve the overall performance of plant genetic resources when tested in a hybrid background.

**FIGURE 3:**
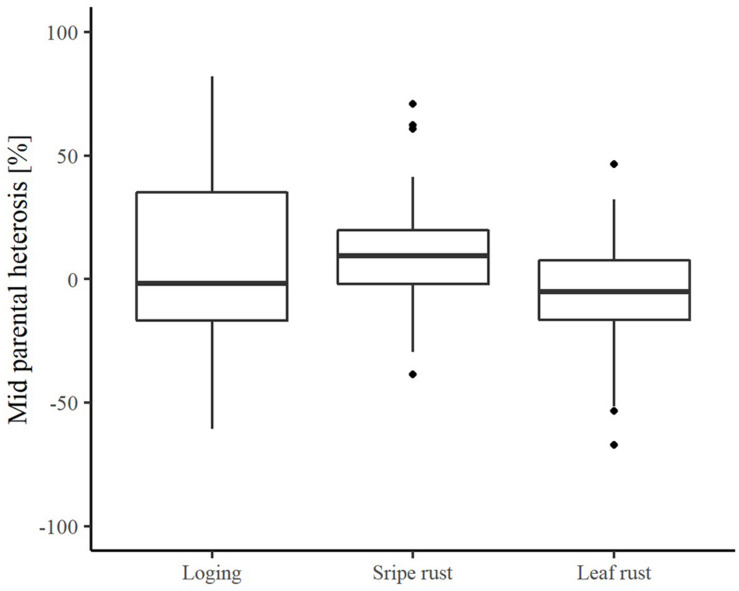
The distribution of relative mid-parent heterosis for the traits lodging tolerance, yellow rust resistance, and leaf rust resistance in hybrid performance experiment.

The average relative mid-parent heterosis (MPH) estimates were −1.83, 9.47, and −5.15%, for lodging tolerance, yellow rust resistance and leaf rust resistance, respectively (Figure 3). The range was quite large with minimum values reaching −60.77% for lodging tolerance, −38.66% for yellow rust resistance, and −67.04% for leaf rust resistance. This clearly underlines the potential to decrease main detrimental effects of plant genetic resources when crossing them with elite lines. Nevertheless, it also highlights the needs to identify appropriate elite tester lines.

**FIGURE 4:**
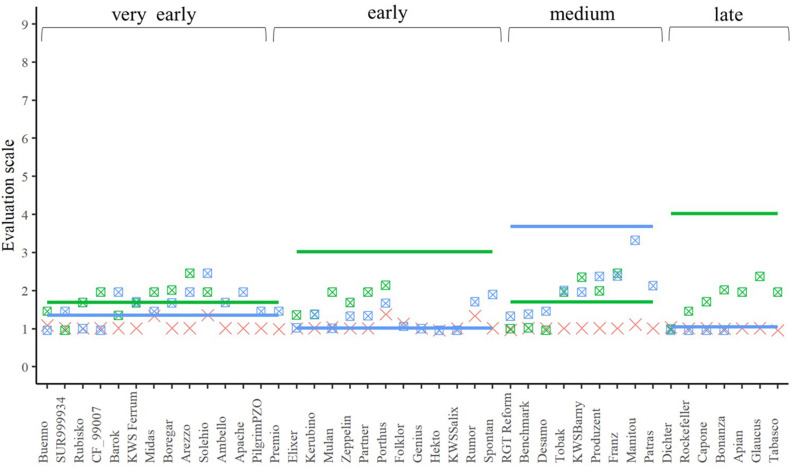
The best linear unbiased estimations of the female (cross), male (line), and their corresponding hybrids (square with cross) for lodging tolerance. The horizontal bars represent the males in each flowering time group. The males and their offspring have the same color.

**FIGURE 5:**
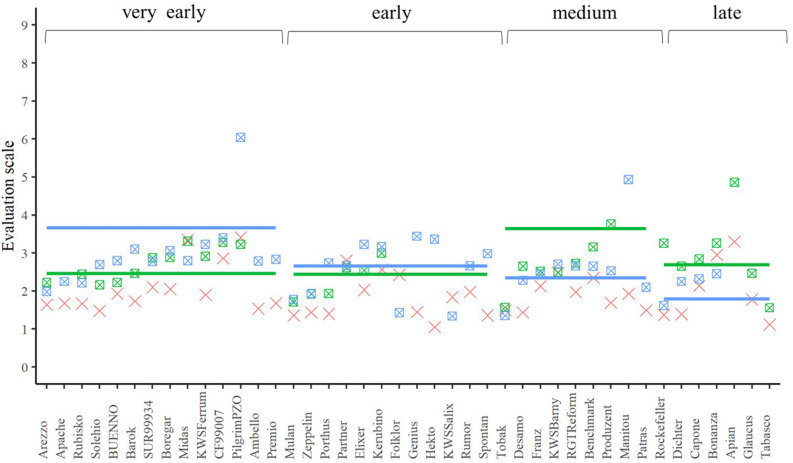
The best linear unbiased estimations of the female (cross), male (line), and their corresponding hybrids (square with cross) for the trait yellow rust resistance. The horizontal bars represent the males in each flowering time group. The males and their offspring have the same color.

**FIGURE 6:**
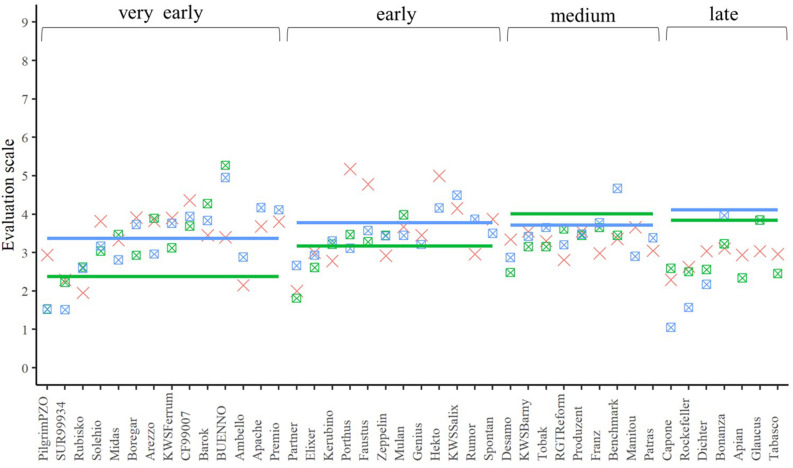
The best linear unbiased estimations of the female (cross), male (line), and their corresponding hybrids (square with cross) for the trait leaf rust resistance. The horizontal bars represent the males in each flowering time group. The males and their offspring have the same color

A closer look at the performance of the single crosses for the three traits (Figures 4–6) underlined that not all genetic resources allowed an equal differentiation of the suitability of female lines as testers. This can be explained by the difficulty of finding excellent pollinators that are simultaneously susceptible to lodging, yellow rust, and leaf rust and must be taken into account when interpreting our results. Despite these shortcomings, our findings revealed that, for example, *Rubisko*, *Elixer*, *Desamo* and *Rockefeller* are suitable elite tester lines that substantially reduce adverse effects of plant genetic resources when tested in a hybrid background.

## Discussion

In order to efficiently evaluate genetic resources of wheat through a hybrid strategy, suitable elite tester lines must be identified that meet the following requirements: The tester lines should (1) possess a high seed yield during hybrid production to provide sufficient multiplicated material for in-depth phenotypic evaluations and (2) reduce detrimental masking effects on grain yield in the hybrid. Information about the latter is scarce. Moreover, previous research has mainly focused on increasing seed yield in hybrid production by optimizing relevant traits of male lines (e.g., Vries, 1971; Whitford et al., 2013; Boeven et al., 2018). In contrast, little is known about the influence of the female lines, which stimulated us to investigate quantitative genetic parameters of seed yield during hybrid production for a set of 63 elite female lines. The hybrid seeds produced were then used to examine the suitability of elite tester lines to reduce masking detrimental effects prevalent in wheat genetic resources.

### Sterility Induced by the Chemical Hybridization Agent Depended on the Genotype

Although the same protocol for the application of the chemical hybridization agent was followed across the years, the variance of environments contributed to 42% of the total phenotypic variance of sterility of the female line (Table 2). This can be explained by a strong influence of weather conditions such as rain, wind or heat on the efficiency of the chemical hybridization agent as noted in previous studies (Pickett, 1993; Kempe and Gils, 2011; Whitford et al., 2013; Easterly et al., 2019). Moreover, the genotype also significantly (*P* < 0.001) contributed to the phenotypic variance of sterility, resulting in a broad-sense heritability of h^2^ = 0.32, which is surprising, since Clofencet, the applied chemical hybridization agent, is expected to act relatively independent of the wheat genotype (Parodi and de los Angeles Gaju, 2009; Nesvadba et al., 2001; Kempe and Gils, 2011; Whitford et al., 2013). At the same time, the interaction between the wheat genotype and the environment influenced the sterility in this experiment. The interaction effects could be related to the variation of nitrogen availability at the different environments (Supplementary Table 2), which may cause pronounced genotypic differences in tillering (Diepenbrock et al., 2009) despite the high sowing density of 300 grains m^–2^ applied in our study. A large number of tillers can make it difficult to apply the chemical hybridization agent at the right developmental stage so that some spikes remain fertile. Consequently, we excluded all non-sterile plots that resulted in a heritability of 0.00 for sterility in the revised data set. This curated data set allows an unbiased estimate of the seed yield in hybrid production.

### The Choice of the Female Tester Lines Impacted the Seed Yield in Hybrid Production

In accordance with previous findings (Vries, 1974; Pickett, 1993; Boeven et al., 2018), large parts (65%) of the differences among the seed yield in hybrid production could be explained by the effect of the environments (Figure 4). Weather probably had the greatest influence in our study (Supplementary Figure 1): Water stress and high temperatures during the season 2017/2018 contributed to a 54% lower seed yield and 11 days earlier flowering compared to the experiments conducted in the other two growing seasons.

The design of the seed production experiment was focused on reducing the general effect of the male lines and the interaction effects between the female and male line by choosing excellent pollinators known from previous hybrid seed production experiments (Boeven et al., 2020). The flowering time synchronization was also considered (Supplementary Figure 2). This approach was successful, and the sum of both variances, i.e., the variance of the effects of the male lines and of the interaction effects between male and female lines, was only half of the variance observed for the effects of the female lines (Table 4). The pronounced effects of the female lines (Figure 2) could be due to differences in pollen-receptivity qualities or to differences in the lines’ *per se* performance. We tested the latter by investigating the association between seed set of the females in the hybrid production and the lines’ *per se* performance determined in previous grain yield trials. The trials included only a subset of 11 female lines, and the comparison showed a correlation coefficient of *r* = 0.39, which indicates that the pronounced variation in female effects is at least partially caused by differences in genuine pollen-receptive qualities. The genuine pollen-receptive qualities of a line might be explained by general structure of the wheat spike: Pickett (1993) reported that spikes with a wide arrangement of spikelets were more easily cross-pollinated than those with a compact spikelet arrangement. Due to a wide arrangement, palea lemma and glumes have a wider opening angle, which means that more foreign pollen reaches the stigma. The length and structure of the stigma can also influence the receptivity (Pickett, 1993; Whitford et al., 2013). A detailed study of the flowering biology during the production of hybrid seed is required to decipher key traits that control pollen-receptivity qualities. The significant contribution of the female lines to the variance in seed yield in hybrid production in our study (Figure 2) highlighted that this is an important undertaking.

**TABLE 4:** Variance components underlying phenotypic variation and broad-sense heritability (h^2^) for the traits plant height, lodging tolerance, resistance to yellow rust and resistance to leaf rust of genotype (σ^2^_*g*_), environment (σ^2^_*e*_), the interaction between the genotype and environment (σ^2^_*g x e*_), and the residual (σ^2^_ε_) in the hybrid performance experiment.

Source	Plant height	Lodging tolerance	Yellow rust resistance	Leaf rust resistance
Number of years	2	2	2	2
Number of environments	2	1	2	2
Number of replication	10.03	3.36	5.27	6.39
σ^2^_*e*_	17.26***	0.00***	1.08***	3.22***
σ^2^_*g*_	100.07***	0.15***	0.17***	0.22***
σ^2^_*g x e*_	30.47***	0.01*	0.28***	0.23***
σ^2^_ε_	16.05	0.18	0.36	1.04
h^2^	0.92	0.71	0.56	0.5

* and *** significantly different from zero with P < 0.05 and 0.001, respectively.

### Reducing Masking Effects of Major Deleterious Traits of Wheat Genetic Resources by Evaluating the Breeding Value in a Hybrid Background

In addition to the seed yield, we also investigated in a second experiment the influence of the choice of female elite tester lines on lodging tolerance and susceptibility to the diseases yellow rust or leaf rust of their hybrids. The tester should be tolerant or resistant and inherit this property to its progenies. Lodging tolerance is influenced by various factors. In addition to the genotype, the weather, the level of the N-fertilization, the use of growth regulators or the infection with eye-spot disease (*Pseudocercosporella herpotrichoides*) can play a role in the phenotypic expression of this trait (Diepenbrock et al., 2009; Obst and Gehring, 2002). Due to the early summer drought in both evaluation years, the trait lodging tolerance was not very pronounced but despite this varieties differed in their expression of the trait (Figure 4). It should be emphasized that the majority of the hybrids showed less lodging than their corresponding male parents, expect for the hybrids based on the male lines, which were not as susceptible (Figure 4). Thus, it can be concluded that the elite female lines can inherit the advantageous trait expression to their progeny – at least partially – and, therefore, the hybrid strategy can be successfully implemented in wheat for lodging tolerance.

Yellow rust and leaf rust are among the most important fungal diseases in wheat (Huerta-Espino et al., 2011; Wellings, 2011) potentially masking the yield potential in field trials. In our field experiment, infection was strongly influenced by the environment (Table 4). This is not surprising, since rust spore germination and uredospore development depend on temperature, humidity, and leaf moisture (McGregor, 1985; Obst and Gehring, 2002). Thus, high temperature and low rainfall in early summer (Supplementary Figure 1) resulted in reduced yellow rust and leaf rust infestations in all experimental years. Despite this we observed significant genetic differences resulting in a minimum mid-parent heterosis of −38.66% and −67.04% for leaf rust and yellow rust resistance, respectively (Figures 5, 6). These findings are concordant with those reported by Longin et al. (2013), clearly indicating that a proper choice of tester reduces the impact of yellow rust and leaf rust susceptibility in hybrid grain yield trials. A proper choice of tester lines may be further supported in the future by a deeper understanding of the degree of dominance at the level of QTL as pointed out by Beukert et al. (2020).

## Conclusion

The choice of suitable female elite tester lines is an important decision to efficiently estimate the breeding value for grain yield of plant genetic resources in hybrid backgrounds. For a stable hybrid seed production with many plant genetic resources, the general combining ability effect of the females for seed yield is a good criterion for selection. Furthermore, suitable female elite tester lines promote the adaption of plant genetic resources to the current arable farming system with regard to the important agronomic traits lodging tolerance and disease resistance in a hybrid background (Sommer et al., 2019). On the other hand, too advanced testers in terms of grain yield can jeopardize the identification of excellent plant genetic resources through the accumulation of dominant positive alleles in the hybrid (Hallauer et al., 1988). This risk, however, is expected to be moderate, because grain yield heterosis in wheat is largely driven by epistasis (Jiang et al., 2017). The results of the two field experiments suggest that several elite lines, such as *Rockefeller*, *Capone*, *Benchmark*, *KWS Barny*, *Elixer*, and *Kerumbio*, are suitable tester lines because of their high grain yield in hybrid production combined with a good ability to reduce detrimental effects in the tested wide crosses.

There are still challenges to be overcome: The advantage of chemical hybridization agents is that they allow a large number of cross combinations without much effort (Whitford et al., 2013). Nevertheless, it has to be taken care to ensure a stable sterility for female lines, is not only critical but also, challenging. Consideration should therefore be given to converting the tester lines into a genetic sterility system. An example would be the “BLue Aleurone (BLA)” system (Darvey, 2019). This would facilitate a sustainable implementation of the hybrid strategy to test the breeding value of wheat genetic resources for grain yield. Moreover, a deeper understanding of the genetics of female receptivity would be an important asset to improve the efficiency of hybrid seed production and, thus, increase the prospects of hybrid wheat breeding.

## Data Availability Statement

The original contributions presented in the study are included in the article/Supplementary Material, further inquiries can be directed to the corresponding author.

## Ethics Statement

All authors declare that this study adheres to ethical standards including ethics committee approval and consent procedure. All experiments were performed under the current laws of Germany.

## Author Contributions

JS, NP, and JR designed the study. JS and MB performed the data analysis. AS advised on the statistical analyses. JS and JR wrote the manuscript. All authors revised the manuscript and agreed with the current statements.

## Conflict of Interest

The authors declare that the research was conducted in the absence of any commercial or financial relationships that could be construed as a potential conflict of interest.
