# Supplementary material for: Assessing the Suitability of Elite Lines for Hybrid Seed Production and as Testers in Wide Crosses With Wheat Genetic Resources
Source: Front Plant Sci. 2021 Jun 14;12:689825. doi: 10.3389/fpls.2021.689825 (PMC8236896; doi:10.3389/fpls.2021.689825)
Supplement: Supplementary file 1 [file Data_Sheet_1.docx]

Supplementary Material

# Supplementary Figures and Tables

## Supplementary Figures


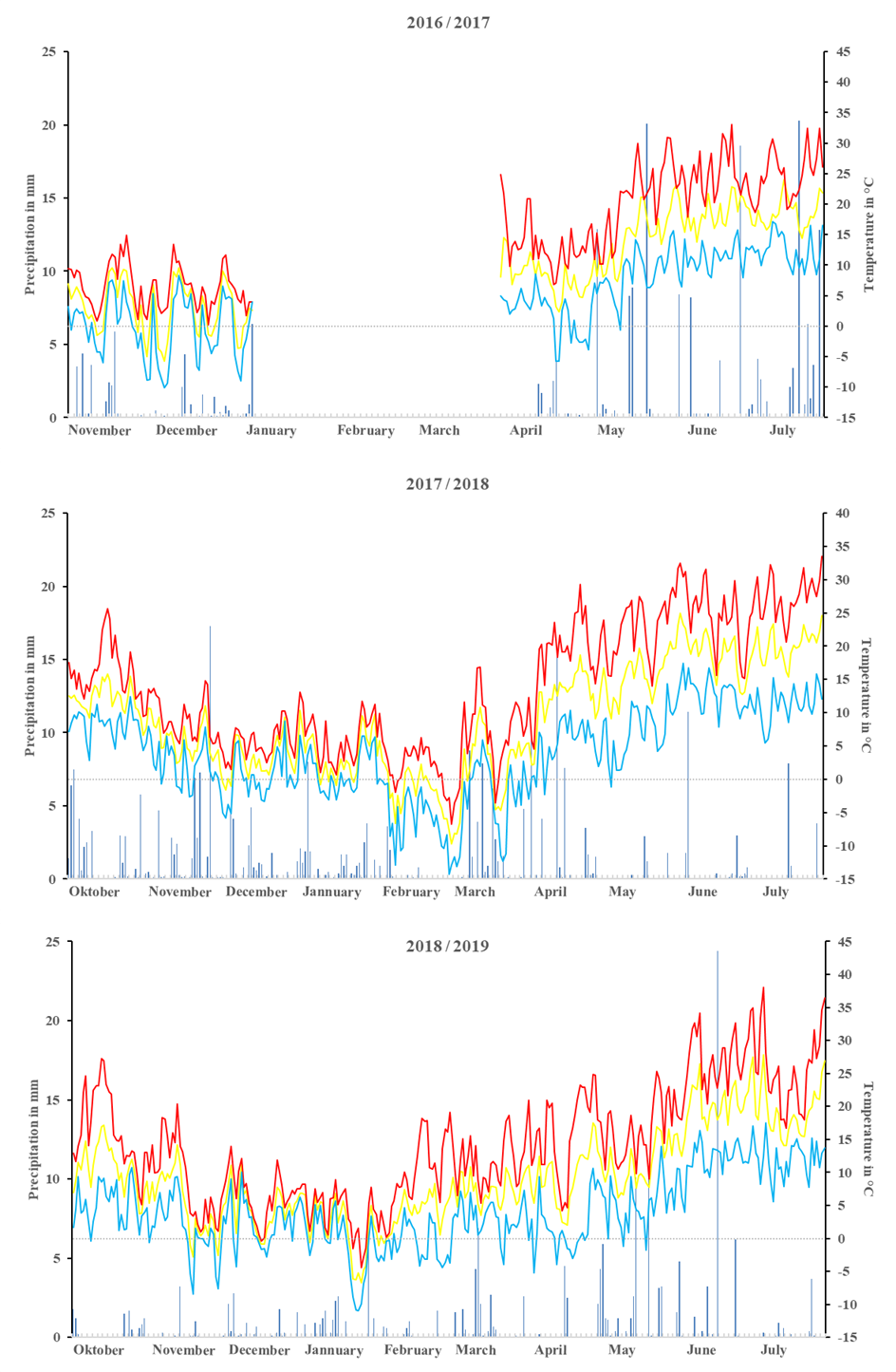


**Supplementary Figure 1 |** Weather data from sowing to harvest of the field trials over the October-July period in the hybrid production experiment. The blue bars represent the amount of precipitation per day in mm. In addition to the daily average temperature (yellow). the maximum (red) and minimum (blue) daily temperatures are shown in the temperature diagram.


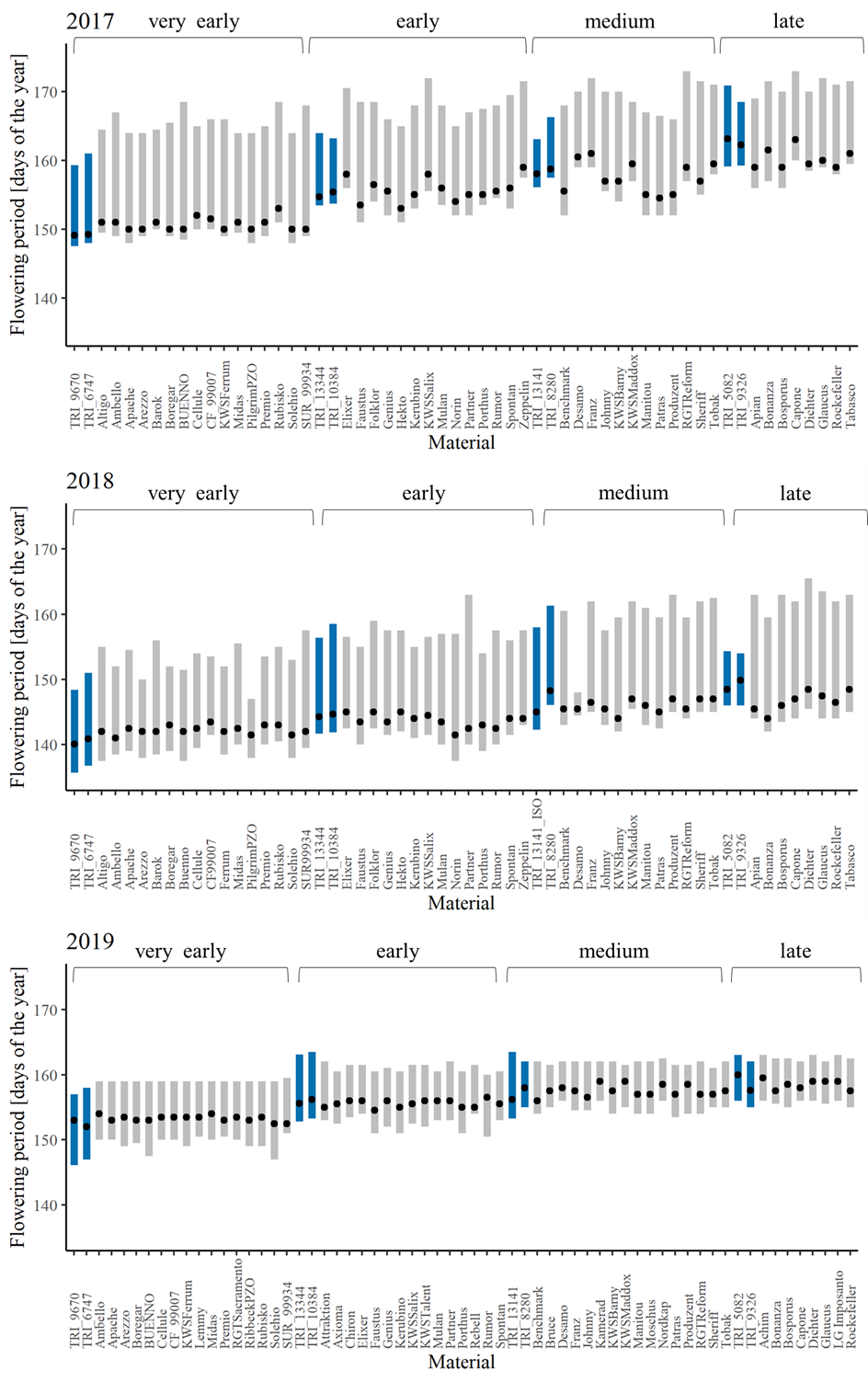


**Supplementary Figure 2 |** The flowering period of the female (grey) and male (blue) lines in the hybrid production experiment. The average flowering periods are shown with bars. The black dot is the time of the main flowering.


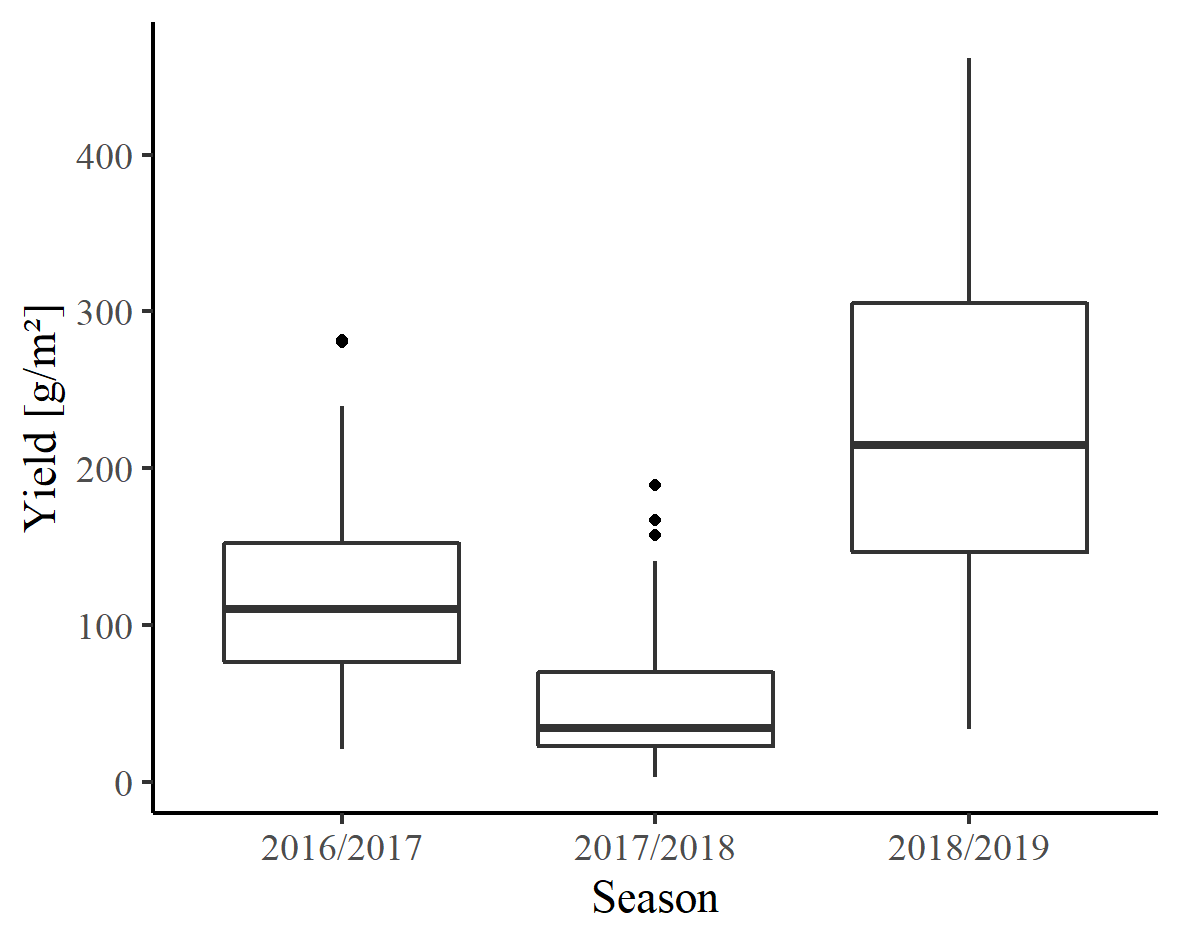


**Supplementary Figure 3 |** The average of the yield on the female in g per m² for the three trial years.

## Supplementary Tables

**Supplementary Table 1 |** Description of elite lines used as female parents in hybrid seed production experiment.

| **Accession** | **Botanical name** | **Flowering time** | **Plant height (cm)** | **Lodging**  **tolerance** | **Yellow rust resistance** | **Leaf rust**  **resistance** |
| --- | --- | --- | --- | --- | --- | --- |
|  |  |  |  |  |  |  |
| Achim | *Trticum aestivum* L. | late | 88.37 | 1 | 2 | 3 |
| Altigo | *Trticum aestivum* L. | very early | - | - | - | - |
| Ambello | *Trticum aestivum* L. | very early | 85.47 | 1 | 2 | 2 |
| Apache | *Trticum aestivum* L. | very early | 81.76 | 1 | 2 | 4 |
| Apian | *Trticum aestivum* L. | late | 81.17 | 1 | 3 | 3 |
| Arezzo | *Trticum aestivum* L. | very early | 82.76 | 1 | 2 | 4 |
| Attraktion | *Trticum aestivum* L. | early | - | - | - | - |
| Axioma | *Trticum aestivum* L. | early | - | - | - | - |
| Barok | *Trticum aestivum* L. | very early | 79.88 | 1 | 2 | 3 |
| Benchmark | *Trticum aestivum* L. | medium | 87.1 | 1 | 2 | 3 |
| Bonanza | *Trticum aestivum* L. | late | 87.57 | 1 | 3 | 3 |
| Boregar | *Trticum aestivum* L. | very early | 82.47 | 1 | 2 | 4 |
| Bosporus | *Trticum aestivum* L. | late | 92.71 | 1 | 1 | 4 |
| Bruce | *Trticum aestivum* L. | medium | - | - | - | - |
| BUENNO | *Trticum aestivum* L. | very early | 76.82 | 1 | 2 | 6 |
| Capone | *Trticum aestivum* L. | late | 81.23 | 1 | 2 | 2 |
| Cellule | *Trticum aestivum* L. | very early | 87.29 | 1 | 2 | 4 |
| CF_99007 | *Triticum aestivum* L. | very early | 76.66 | 1 | 3 | 4 |
| Chiron | *Trticum aestivum* L. | early | - | - | - | - |
| Desamo | *Trticum aestivum* L. | medium | 82.28 | 1 | 1 | 3 |
| Dichter | *Trticum aestivum* L. | late | 79.89 | 1 | 1 | 3 |
| Elixer | *Trticum aestivum* L. | early | 91.05 | 1 | 2 | 3 |
| Faustus | *Trticum aestivum* L. | early | 93.63 | 1 | 1 | 5 |
| Folklor | *Trticum aestivum* L. | early | 88.97 | 1 | 2 | 3 |
| Franz | *Trticum aestivum* L. | medium | 91.68 | 1 | 2 | 3 |
| Genius | *Triticum aestivum* L. | early | 91.06 | 1 | 1 | 3 |
| Glaucus | *Trticum aestivum* L. | late | 90.34 | 1 | 2 | 3 |
| Hekto | *Trticum aestivum* L. | early | 79.68 | 1 | 1 | 5 |
| Johnny | *Trticum aestivum* L. | medium | 89.47 | 1 | 3 | 3 |
| Kamerad | *Trticum aestivum* L. | medium | - | - | - | - |
| Kerubino | *Trticum aestivum* L. | early | 88.82 | 1 | 3 | 3 |
| KWSBarny | *Trticum aestivum* L. | medium | 83.62 | 1 | 2 | 4 |
| KWSFerrum | *Triticum aestivum* L. | very early | 85.13 | 1 | 2 | 4 |
| KWSMaddox | *Trticum aestivum* L. | medium | 88.67 | 1 | 2 | 3 |
| KWSSalix | *Triticum aestivum* L. | early | 94.87 | 1 | 2 | 4 |
| KWSTalent | *Trticum aestivum* L. | early | - | - | - | - |
| Lemmy | *Trticum aestivum* L. | very early | - | - | - | - |
| LG Imposanto | *Trticum aestivum* L. | late | - | - | - | - |
| Manitou | *Trticum aestivum* L. | medium | 89.21 | 1 | 2 | 4 |
| Midas | *Trticum aestivum* L. | very early | 99.67 | 1 | 3 | 3 |
| Moschus | *Trticum aestivum* L. | medium | - | - | - | - |
| Mulan | *Trticum aestivum* L. | early | 91.38 | 1 | 1 | 4 |
| Nordkap | *Trticum aestivum* L. | medium | - | - | - | - |
| Norin | *Trticum aestivum* L. | early | 87.23 | 1 | 2 | 3 |
| Partner | *Trticum aestivum* L. | early | 90.68 | 1 | 3 | 2 |
| Patras | *Triticum aestivum* L. | medium | 89.53 | 1 | 1 | 3 |
| Pilgrim PZO | *Trticum aestivum* L. | very early | 88.53 | 1 | 3 | 3 |
| Porthus | *Trticum aestivum* L. | early | 93.96 | 1 | 1 | 5 |
| Premio | *Trticum aestivum* L. | very early | 79.61 | 1 | 2 | 4 |
| Produzent | *Trticum aestivum* L. | medium | 87.87 | 1 | 2 | 4 |
| Rebell | *Trticum aestivum* L. | early | - | - | - | - |
| RGTReform | *Triticum aestivum* L. | medium | 82.09 | 1 | 2 | 3 |
| RGTSacramento | *Trticum aestivum* L. | very early | - | - | - | - |
| RibbeckPZO | *Trticum aestivum* L. | very early | - | - | - | - |
| Rockefeller | *Trticum aestivum* L. | late | 86.93 | 1 | 1 | 3 |
| Rubisko | *Trticum aestivum* L. | very early | 80.15 | 1 | 2 | 2 |
| Rumor | *Trticum aestivum* L. | early | 93.68 | 1 | 2 | 3 |
| Sheriff | *Trticum aestivum* L. | medium | - | - | - | - |
| Solehio | *Trticum aestivum* L. | very early | 86.35 | 1 | 1 | 4 |
| Spontan | *Trticum aestivum* L. | early | 91.98 | 1 | 1 | 4 |
| SUR_99934 | *Triticum aestivum* L. | very early | 86.51 | 1 | 2 | 2 |
| Tabasco | *Triticum aestivum* L. | late | 78.18 | 1 | 1 | 3 |
| Tobak | *Trticum aestivum* L. | medium | 87.97 | 1 | 1 | 3 |
| Zeppelin | *Trticum aestivum* L. | early | 90.52 | 1 | 1 | 3 |

**Supplementary Table 2 |** Characterization of the three environments of the hybrid seed production experiment.

|  | **Year** | | |
| --- | --- | --- | --- |
|  | **2017** | **2018** | **2019** |
| Location | 51°49'55.6"N 11°16'15.3"E | 51°49'37.1"N 11°16'09.7"E | 51°48'26.6"N 11°15'17.9"E |
| Soil type | tL | Lt | uL |
| pH (CaCl_2_)^a^ | 7.30 | 7.50 | 6.10 |
| P (CAL) (mg/ 100g ^-1^ )^a^ | 14.20 | 31.20 | 3.40 |
| K_2_O (mg /100 g ^-1^ )^a^ | 17.20 | 18.70 | 18.80 |
| Nmin-total (kg ha ^-1^)^b^ | 138.00 | 162.00 | 211.00 |

^a^ soil depth 0- 30 cm

^b^ soil depth 0- 60 cm

**Supplementary Table 3 |** The correlation of hybrid seed yield between the three trial years. The number of linkage varieties is listed on the bottom of the diagonal.

|  | **2016/2017** | **2017/2018** | **2018/2019** |
| --- | --- | --- | --- |
| **2016/2017** | - | 0.63^***^ | 0.58^**^ |
| **2017/2018** | 35 | - | 0.81^***^ |
| **2018/2019** | 28 | 29 | - |

**Supplementary Table 4 |** Variance components underlying phenotypic variation of plant height, lodging, yellow rust, and leaf rust in the hybrid performance experiment. Phenotypic variation was decomposed into environment(σ²_e_), female (σ²_f_), male (σ²_m_) their interaction (σ²_f x m_, σ²_f x e_ σ²_m x e_) and the residual (σ²_ε_).

| **Source** | **Plant height** | **Lodging**  **tolerance** | **Yellow rust resistance** | **Leaf rust resistance** |
| --- | --- | --- | --- | --- |
| No. of Year | 2 | 2 | 2 | 2 |
| No. of Environments | 2 | 1 | 2 | 2 |
| No. of Replication | 10.3 | 3.36 | 5.27 | 6.39 |
| σ²_e_ | 18.01 | 0.01 | 0.99 | 3.15 |
| σ²_f_ | 3.89 | 0.06 | 0.14 | 0.18 |
| σ²_m_ | 447.99 | 0.17 | 0.16 | 0.00 |
| σ²_f x m_ | 5.53 | 0.00 | 0.00 | 0.00 |
| σ²_f x e_ | 0.00 | 0.01 | 0.10 | 0.02 |
| σ²_m x e_ | 22.75 | 0.00 | 0.06 | 0.08 |
| σ²_ε_ | 108.48 | 0.34 | 0.70 | 1.42 |
